# Health Care Robotics: Qualitative Exploration of Key Challenges and Future Directions

**DOI:** 10.2196/10410

**Published:** 2018-07-04

**Authors:** Kathrin Cresswell, Sarah Cunningham-Burley, Aziz Sheikh

**Affiliations:** ^1^ Usher Institute of Population Health Sciences and Informatics University of Edinburgh Edinburgh United Kingdom

**Keywords:** robotics, health care, sociotechnical

## Abstract

**Background:**

The emergence of robotics is transforming industries around the world. Robot technologies are evolving exponentially, particularly as they converge with other functionalities such as artificial intelligence to learn from their environment, from each other, and from humans.

**Objective:**

The goal of the research was to understand the emerging role of robotics in health care and identify existing and likely future challenges to maximize the benefits associated with robotics and related convergent technologies.

**Methods:**

We conducted qualitative semistructured one-to-one interviews exploring the role of robotic applications in health care contexts. Using purposive sampling, we identified a diverse range of stakeholders involved in conceiving, procuring, developing, and using robotics in a range of national and international health care settings. Interviews were digitally recorded, transcribed verbatim, and analyzed thematically, supported by NVivo 10 (QSR International) software. Theoretically, this work was informed by the sociotechnical perspective, where social and technical systems are understood as being interdependent.

**Results:**

We conducted 21 interviews and these accounts suggested that there are significant opportunities for improving the safety, quality, and efficiency of health care through robotics, but our analysis identified 4 major barriers that need to be effectively negotiated to realize these: (1) no clear pull from professionals and patients, (2) appearance of robots and associated expectations and concerns, (3) disruption of the way work is organized and distributed, and (4) new ethical and legal challenges requiring flexible liability and ethical frameworks.

**Conclusions:**

Sociotechnical challenges associated with the effective integration of robotic applications in health care settings are likely to be significant, particularly for patient-facing functions. These need to be identified and addressed for effective innovation and adoption.

## Introduction

**Figure 1:**
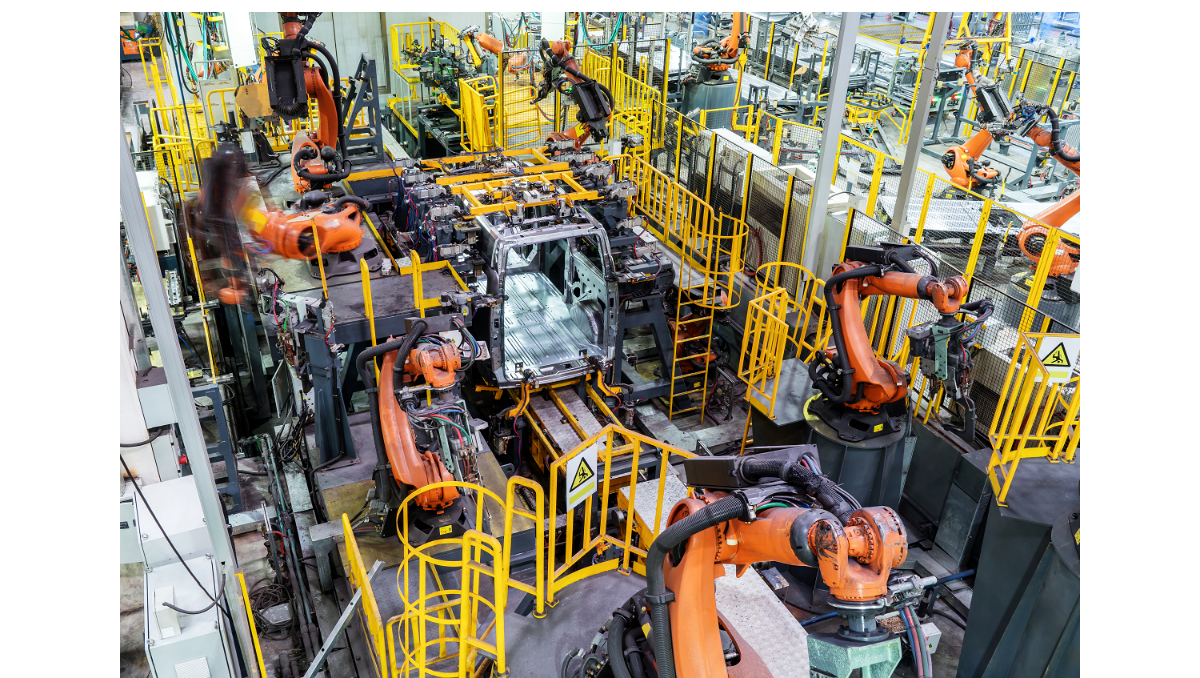
Robotics in car manufacturing. Source: gyn9037/Shutterstock.com.

We are amid what has been described as the Fourth Industrial Revolution, where industries and sectors across the globe are being transformed using a variety of increasingly interconnected robotic applications [1]. These have demonstrably increased productivity, resource efficiency, and customer responsiveness in, for example, the manufacturing and retail sectors (see Figure 1) [2,3]. Amazon, for instance, now has a 100,000-robot fleet designed to navigate large warehouse spaces and pick items from shelves. This represents a 50% increase from the previous year such that robots now constitute around one-third of the workforce [4,5].

**Figure 2:**
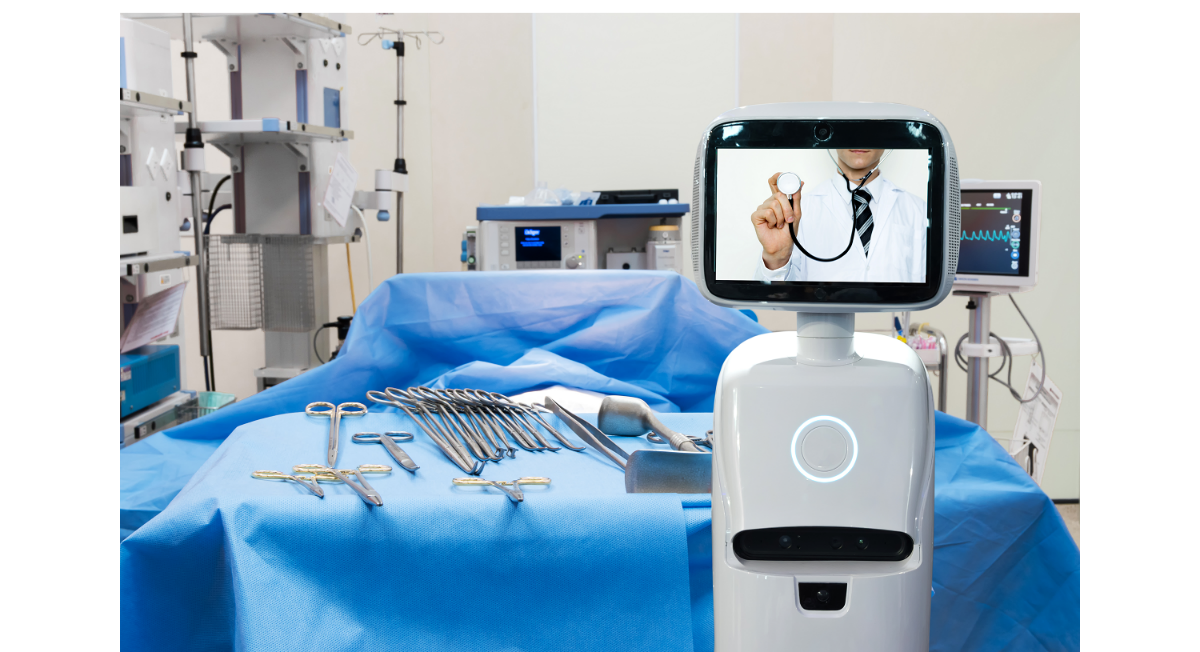
Robotics in health care. Source: Zapp2Photo/Shutterstock.com.

There is emerging policy interest in seeing a similar transition in health care; this is being fueled by the drive to improve the quality and safety of care while simultaneously controlling expenditure [6]. Developments currently taking place have begun to replace individual aspects of human performance with robotic capabilities including precision (eg, surgical robots), logistic and mechanical tasks (eg, service robots), and complex cognitive tasks (eg, rehabilitation robots; see Figure 2 and Table 1) [7].

Deployments of robots in health care settings are likely to rise because of increasing technological capabilities, their reduced costs, and increasing pressure to curb costs. However, robots are potentially highly disruptive innovations, and it is therefore important to understand the sociotechnical challenges likely to be encountered as robots are deployed to find mitigating strategies [8-10]. Sociotechnical approaches to study the implementation of technology view social and technical factors as shaping each other over time. It is assumed that technologies are shaped by their social environments (eg, through designs being modified) but also that social environments are shaped by technological features (eg, when work practices of users change as a result of technology introduction).

**Table 1:** Uses of health care robotics.

Type of device	Autonomous	Semiautonomous	Operational	Health care delivery, patient- and staff-facing
Service robots (eg, stock control, cleaning, delivery, sterilization)	✓	✓	✓	
Surgical robots		✓		✓
Telepresence robots (eg, screens on wheels)		✓		✓
Companion robots	✓			✓
Cognitive therapy robots	✓			✓
Robotic limbs and exoskeletons		✓		✓
Humanoids	✓		✓	✓

Such insights can support the development of an informed robotics strategy for health care that addresses these upcoming challenges (eg, by training staff and designing existing spaces appropriately), thus supporting the aim of transformation of health care through health information technology (HIT). To inform these important deliberations, we undertook an exploratory qualitative study to identify key sociotechnical challenges associated with introducing robotics in health care settings from the standpoint of key stakeholders.

## Methods

### Overview

We conducted an interview-based qualitative case study consulting stakeholders from various backgrounds and disciplines [11]. In doing so, health care robotics was conceptualized as the case. Other case studies currently in progress as part of a wider project exploring next generation technologies in health care settings include the integration of patient- and person-generated data with electronic health records (EHRs), innovative information infrastructures, and novel approaches to secondary data analysis.

### Ethics and Permissions

This study received Institutional Review Board approval from the Centre of Population Health Sciences at the University of Edinburgh, United Kingdom. Participants gave written informed consent to participate, and transcripts were anonymized.

### Sampling and Recruitment

Participants were sampled through Google searches using search terms relating to robotics and health care. We sampled purposefully for maximum variability ensuring presentation from a range of countries and professional backgrounds (including engineers, system developers, suppliers, academics, visionaries/futurists, users of robots in health care settings, and strategists) [12]. In line with the sociotechnical approach, the range of perspectives was expected to give important insights into the technical and social environments of robotic applications in health care settings. This sampling strategy was complemented by snowball sampling additional participants [13]. As our purpose was to develop a high-level overview, we did not specifically sample for individual users of applications in specific contexts.

Overall, we identified 68 participants. Of these, 42 were contacted through publicly available email addresses. The rest were sent invitations via LinkedIn through the account of the first author (KC). The initial email included an invitation to participate and an overview of the work. If participants expressed an interest (17/68 did), they were sent an information sheet and consent form. The remainder were sent a follow-up email approximately 2 weeks later (resulting in 9 additional responses). After initial discussions, 5 potential participants decided not to participate, mainly due to concerns surrounding signing the consent form and the interview being audio-recorded (although an option of not recording was offered). Industry representatives were not comfortable sharing potentially sensitive commercial information.

### Data Collection

Interviews were conducted over Skype, digitally recorded, and transcribed verbatim by a professional transcriber. These ranged from 30 to 90 minutes, depending on the schedule of the participant and the number of issues they wanted to discuss. We explored the most promising areas surrounding health care robotics, their benefits and risks, anticipated and observed challenges, and potential ways to address these from a variety of technical and social angles. A sample interview guide can be viewed in Textbox 1.

We stopped recruiting participants when we reached thematic saturation (ie, when no new themes emerged during the concurrent analysis) [14]. To ensure that participant voices were reflected accurately, we performed member checking by sending the results to all participants and giving them the opportunity to comment on and correct any misunderstandings [15]. This resulted in minor clarifications to the results, consisting mainly of adding further details and context.

### Data Analysis

Transcribed interviews were uploaded to NVivo 10 (QSR International Pty Ltd) software, which supports the management and interrogation of data and helps arrange qualitative data into meaningful headings and subheadings. We began the coding process as soon as interviews were transcribed to allow emerging findings to feed into future interviews; this involved sorting data into meaningful headings and subheadings for ongoing thematic analysis.

**Sample interview topic guide.**

**Vision surrounding robotics and automation in health care:**

- Most promising developments to look out for, benefits
- What processes lend themselves best to automation?
- Any risks, issues that are particularly relevant to robotics
- Convergence of robotics, artificial intelligence, and big data analytics: how is the area of robotics defined?

**Experiences of technological innovation in health care:**

- Experiences and lessons learned
- User involvement in design
- Anything we can learn from other sectors?
- Which factors hinder developments, and how might these be addressed?

We approached the analysis with an initial coding framework based on the available empirical literature surrounding sociotechnical factors of technology implementation in health care settings [16]. The deductive components were as follows:

- Technological dimension (including technological features, technological infrastructures)
- Social/human dimension (including usability, human-technology interaction, attitudes)
- Organizational dimension (including organizational strategy, management, implementation)
- Macro-environmental dimension (regulation, legal, and ethical dimensions)

This allowed us to provide initial structure to our findings that remained close to the research question and sociotechnical perspective underpinning it. The coding framework was informed by our previous theoretical work surrounding the evaluation of sociotechnical systems [17]. In addition, we allowed new themes to emerge based on the frequency of occurrence and perceived significance (inductive component). During this process, we explored disconfirming evidence and carefully questioned our own (in some instances critical) assumptions about robotics.

In doing so, we carefully compared technological features, participant backgrounds, and insights into various sociotechnical aspects surrounding conceptualization, design, implementation, and adoption of technologies. Emerging themes were discussed and refined during regular meetings between the authors, paying particular attention to the intersection of technical and social factors in line with the sociotechnical lens.

## Results

### Overview

We interviewed a total of 21 participants (see Table 2). They came from a range of countries and academic, industry, and strategic backgrounds. Some, particularly academics, had mixed clinical backgrounds and had used or investigated robotic applications in health care contexts.

We identified a range of themes and subthemes, summarized in Textbox 2.

Overall, participants stated that the area of robotics in health care settings was still in its infancy and the move from paper-based to EHRs currently took strategic priority over investments in robotics. Specifically, the more novel developments surrounding humanoids were still seen to be a long way off in terms of routine deployment in health and care settings, while service robots were seen to hold the biggest short-term promise. However, it was also acknowledged that there was significant potential and the pace of developments as well as increasing convergence of applications meant that robotics was likely to become a routine aspect of health care delivery at some point.

I am quite taken by the fact of how quickly changes come about...in my lifetime as a surgeon in the late ’80s we completely switched over a 2-year period from an open surgical approach to a minimal and key hole...

Participant 2, surgeon, United States, male

While some of the issues identified applied to all robotic uses outlined in Table 1, we also observed a hierarchy of features with increasing levels of sociotechnical complexity. For instance, semiautonomous operational applications tended to be viewed as presenting fewer sociotechnical challenges than autonomous care-facing functions. Further, there were subtle differences between participants from different backgrounds, with academics and strategists being slightly more critical, citing a wider range of challenges than commercial participants.

### No Clear Pull From Professionals and Patients

There was a perception that concerns among the public, patients, and health care staff could hold back progress, leading to a lack of demand or acceptance for some robotic applications in health care settings. Attitudes were seen to be heavily influenced by negative publicity and modern science fiction.

...[patients] think when you say robot...you mean Terminator, so people are afraid...

Participant 8, technologist, France, male

Such negative attitudes were seen to be due to a range of factors. Some mentioned the clinician-patient relationship and patient trust as aspects of care that were perceived to require human input. Therefore, applications seen to be performing tasks of a health care professional were viewed as particularly contested.

**Table 2:** Participant characteristics.

Participant number	Background	Country	Gender
1	Marketing: service robots	United States	Female
2	Surgeon: user of surgical robots	United States	Male
3	Academic: research into service robots	Norway	Male
4	Engineer: surgical robots	United States	Male
5	Futurist	United States	Female
6	Marketing: sterilization robots	Italy	Female
7	Academic, sociotechnical perspective	Switzerland	Male
8	Technologist: humanoids	France	Male
9	Academic: mainly surgical robots	United States	Male
10	Technologist	United Kingdom	Male
11	Engineer: telepresence robots	United States	Male
12	Academic	Sweden	Female
13	Strategist	Netherlands	Male
14	Journalist	United States	Male
15	Information technology consultant	United States	Male
16	Academic: informatics, rehabilitation, and surgical robots	United Kingdom	Male
17	Business development: humanoids	France	Male
18	Manager, robotics organization	France	Male
19	Academic: surgical robots	Australia	Male
20	Academic, ethicist	United Kingdom	Female
21	Academic, psychologist	United Kingdom	Female

**Themes identified in our work.**

**No clear pull from professionals and patients:**

- Robots have negative publicity
- Lack of acceptance: trust is a social phenomenon and essential for health care
- Robots are transcending the human-machine interaction
- Lack of exposure to robots, particularly in Western cultures

**Appearance of robots:**

- Too robotic: psychological association with death, Terminator movie, fear of replacing human being
- Too human: expectations too high

**Changes to the way health care work is organized and distributed:**

- Changes to roles (replacing human capabilities versus augmenting them)
- Changes to workflows

**New ethical and legal challenges:**

- No existing liability and ethical frameworks
- Anticipating challenges will be crucial in the future
- Regulation is key to promote routine use

Purely service-based robots carrying out back-end functions were often seen as better suited to automation.

...to put your trust into a robot is still not there. I think a walker with robotic features is easier to adopt in the market by the people using it than a lifting robot.

Participant 13, strategist, Netherlands, male

From a health care provider point of view, negative attitudes were seen to be influenced by perceived threats to professional roles.

...a good anesthesiologist [costs] about $350 per hour, it’s a heck of a wage, and the machine can be rented for about $150 so it’s a lot more cost effective. That company have abandoned the product, not because it didn’t function, it functioned extremely well. But it was very unpopular, and it had all sorts of doctor, patient unions and lobbying groups that had kittens about this idea of this robot that could basically put them all out of a job.

Participant 5, futurist, United States, female

Lack of exposure to robots was a major barrier to developing positive attitudes among patients and staff. This was seen to be since many existing applications such as pharmacy robots mainly operated in the back office, and there was a resulting fear of the unknown, particularly in Western cultures where robots are not routinely embedded in other aspects of everyday life.

...in Japan people believe that robots also have a kind of soul and that’s why they approach robots as if they are like normal people. I believe in the rest of the world probably people...will be much more skeptical and I don’t believe that people will accept that particularly caring for people will be performed by robots.

Participant 7, academic, Switzerland, male

Some participants suggested that public engagement campaigns, training of health care staff, and public dissemination of positive robotic case studies could help promote positive attitudes and acceptance of robotic applications among health care staff and patients.

### Appearance of Robots

Humanoids presented a particularly interesting illustration of the tension between human hopes and expectations of robots and apprehension of their use in health care settings. They also represent an important sociotechnical example as human and technical dimensions blur in challenging and highly visible ways.

One reason identified in the interviews for humanoids not being very successfully integrated within health care settings was the contested nature of robotic appearances. On one hand, human features were seen as desirable in order to provide patients and staff with an experience of care as close to the real thing as possible.

We’ve tried to make it as approachable and friendly looking as possible because some people might think it’s cold and now you’re not having a direct person to person interaction in the flesh. So, we try to do our best to really make it as close as possible to the person being there, you know, with good audio, good video, the physical look of the robot.

Participant 11, engineer, United States, male

On the other hand, if robots were designed too human-like, there was significant apprehension of users reported, potentially being due to a fear of the robot replacing humans and imagined parallels with the Terminator vision of robots. This was particularly true for intimate tasks that often represented important aspects of the patient-provider relationship.

...there’s a fear that the robot becomes almost like a near-human doppelganger that replaces the human being, because it has capabilities that we don’t, so there’s still this almost like mythical status of the robot that’s certainly something that hovers around popular consciousness.

Participant 21, manager, United Kingdom, female

An additional undesired consequence was that when robots were designed as too human-like, they often fell short of human expectations of what they could do, resulting in disappointment and lack of engagement with and trust of the robot if it did not perform as expected.

...your expectations go up when you make robots human like.

Participant 16, academic, United Kingdom, male

Some also mentioned that the difficulty of placing humanoid robots firmly within either human or robotic categories was responsible for potential feelings of aversion. This was further exacerbated by a struggle to establish whether to perceive the robot as a friend or foe.

To address these problems, developers tended to design humanoid robots intentionally as non–human-like to ensure a visible demarcation between human and robotic features. This included, for example, designing them to roll on wheels rather than having legs or by designing them in the shape of an animal that most people had no experience interacting with (eg, a baby seal). This strategy was considered to be successful in promoting acceptability across contexts.

### Disruption of the Way Work is Organized and Distributed

All participants acknowledged that the integration of robotic applications with existing health care professional work practices was important but difficult to achieve due to tensions between standardization through automation and the often-unpredictable nature of health care profession work.

The design of robotic applications that interacted with humans and spanned departmental and professional boundaries (ie, as autonomous robots) was seen as particularly problematic, as these transcended capabilities that were previously situated firmly within the human realm (eg, moving around, emotional support).

...when it comes into practice we all ran into problems. What if an elderly person is moving away from a robot, can it follow the elderly person? What if [the robot] falls, and it’s a person with mild dementia. Is that person able to put the robot back on its feet again?

Participant 13, strategist, Netherlands, male

In contrast, robotic applications that were designated for particular uses (such as surgery, where they basically represented a sophisticated tool) or confined to back-office functions in controlled environments (such as pharmacy robots) were seen as less difficult to implement, as they had fewer challenging sociotechnical implications.

...this is why robots have been so successful in industry, like in car manufacturing, because they have these repetitive tasks and there are no humans in their way, they don’t have to make decisions, they don’t have to understand anything.

Participant 14, journalist, United States, male

It was therefore argued that to promote integration, robots should be viewed as augmenting human capabilities and empowering professionals in their role.

...when people talk about nurses and doctors and automation in a hospital, for example, the automation isn’t about replacing the nurses and doctors, it’s about augmenting their role so that they’re more efficient so that they’re not doing endless amounts of paperwork...they spend a bit more time with patients.

Participant 10, technologist, United Kingdom, male

This would, however, require some shift in skill sets toward supporting robotic capabilities and functions, particularly for lower skilled tasks. Envisioning and anticipating those changes was viewed as an important activity for educators, decision makers, and managers in health care settings.

### New Ethical and Legal Challenges

Robotic applications engender new ethical and legal challenges surrounding their use in highly human social settings, and interviewees gave many examples. Some of these tackled the physical environment.

There was one lady who got trapped in an elevator together with one of the robots, and another one got run over.

Participant 3, academic, Norway, male

Others described psychological challenges such as the perceived risk of becoming too emotionally attached to a robot (particularly in care settings where patients are vulnerable).

...if you look at the target audience this will be vulnerable people, disabled people, sick people, the elderly...so it’s important that we have robots that do not transport a feeling that is not real, like companionship robots, for example. They should be designed in a way that it’s always clear that it’s always a robot and not a substitute for a human.

Participant 20, academic, United Kingdom, female

Ethical dimensions surrounding nonuse of technology were also mentioned. These included issues of whether health care professionals should be forced to use a robotic application if this were a safer alternative than human-delivered care.

I’m particularly trying to answer the questions like if we show that you can do something more safely with the robot does that mean that people should use the robots if they know there’s a safer alternative...should they be forced to use a robot assistant because they know it’s a safer way of doing it...

Participant 19, academic, Australia, male

Some had begun developing ethical frameworks for robotic applications. A defining characteristic was that both human and machine perspectives were represented so that the guiding principles were both machine logic and human logic (including their reaction to machine behavior), implying that a new sociotechnical approach to HIT ethics is developing.

...the idea is that the framework is understandable by both humans and machines so that if a machine needs guidance, a human can work through the framework and figure out where it got stuck and make a judgment call or vice versa. Machines can begin to understand how humans themselves are making a certain decision and provide guidance or insight into that.

Participant 5, futurist, United States, female

Additionally, interviewees noted that there was a lack of clear, established liability rules surrounding robotics, made all the more problematic given the perceived hype surrounding robotics and a certain keenness of getting these into use quickly. This meant that when accidents happened (such as robots running over humans), these often had to be solved ad hoc, further contributing to negative public attitudes and inhibiting innovation.

Participants suggested that a more deliberative approach was needed to create clear liability rules surrounding product and consumer safety across different settings in which robotic applications were used, including health care. However, it was seen to be crucial to find a balance between developing overarching rules and allowing innovation to flourish.

## Discussion

### Principal Findings

Although there has been substantial technological progress in the field of health care robotics, robot integration into health care settings is likely to be far from straightforward. We have identified several concerns that are often shaped by preconceptions surrounding the appearance of robotic applications and associated (often conflicting) desires for human and technological features. In addition to these negative attitudes that result in a lack of user pull and demand, robotics also does and will change the way health care work is organized and distributed with some applications augmenting and others replacing human labor. These changes require new ethical and liability frameworks as new situations may emerge that blur the line between human responsibility and technological autonomy.

### Comparison With Prior Work

In undertaking this study, we have elicited the perspectives and experiences of stakeholders from various international settings to bring together knowledge and deliberate on potential future challenges of implementing and optimizing robotic applications in health care settings. We have identified sociotechnical challenges associated with various technological features. This builds on previous work focused on specific systems already being used in specific settings [18-21]. Our focus, in line with our uses (Table 1), was on different aspects of robotic hardware function. Although these were necessarily combined with some software capability including artificial intelligence, software was not the focus of our work.

There is an increasing recognition that sociotechnical considerations are important when considering technological applications including robotics [22,23] but only a limited number of studies have examined such issues with regard to robotic applications in health care [24-26]. Where it exists, primary research has concentrated on technologies in specific environments, including some in health care [27,28]. However, when compared to other HIT, autonomous applications (such as humanoids) present specific sociotechnical challenges because social and technical dimensions are progressively, visibly, and disruptively interconnected. As a result, there is a danger that these sociotechnical challenges will lead to an increasing range of problems integrating robotic applications within particularly human-dense social environments such as health care.

Ethical dimensions surrounding robotics, especially relating to trust and acceptance, have received relatively high levels of attention, perhaps due to perceived negative public attitudes surrounding robotic systems [29-31]. Our work has supported existing research highlighting that these issues pose important sociotechnical barriers to progress. Humans must renegotiate their roles within increasingly technological environments, and this negotiation is characterized by a conceptual struggle between a desire for progress and an apprehension toward the increasingly human side of machines.

Although important as a subject of ongoing debate, these issues are unlikely to ever be fully resolved. Some have found that trust and positive attitudes toward robotic applications can be promoted through exposure [28,32], and exposure is likely to be key in going forward. As robotic applications become more visible in everyday environments, they are likely to become more acceptable in health care settings. Lack of exposure is likely to be a transient issue as there are now many examples in other industries and countries where robots and humans routinely work alongside each other.

### Limitations

The response rate to interview invitations was low (only 21 out of 68 individuals agreed to be interviewed), in part reflecting concerns about disclosing commercially sensitive information. We may therefore have missed some important considerations (despite having achieved thematic saturation within our sample), particularly from cultures that have integrated robotics in everyday life (eg, Japan). Additional factors that are likely to have shaped the sampling of respondents include the presence on Google and LinkedIn, access to Skype, English language facilities, and the Google search methods employed by the researcher. We therefore necessarily explored the views of those who were visible and vocal in relation to health care robotics in English media. Although this was appropriate for gaining a high-level overview into an underexplored topic, it also means that our results are likely to have missed the perspectives of certain user groups (eg, health care professionals and patients with or without the experience of robotics). This may have led to a lack of insight into the acceptability of specific applications. Such work is important going forward as many of the challenges identified are heavily dependent on individual settings, technologies, and contexts. Moreover, we acknowledge that we have only skimmed the surface of exploring ethical, legal, and policy dimensions of robotic applications in health care settings, and this would certainly be a fruitful area for further in-depth research. There was also a clear gender imbalance toward male respondents in our sample, perhaps due to the fact that experts in this area are predominantly male.

### Implications for Research, Policy, and Practice

We have begun charting the range of sociotechnical challenges that are likely to test the routine integration and optimization of robotics into health care settings. We summarize these along with possible ways to address them in Table 3.

Although there is a large literature base addressing the promises of robotics, this is limited to applications other than health care or specific health care applications such as surgery [1,33]. There is a need for empirical investigations into potential challenges and unintended consequences of such technologies in health care settings.

New ethical and regulatory frameworks are now needed that are nimble enough to keep up with changing environments and the increase in and convergence of robotic functionality. This may need to involve training a new generation of professionals who specialize in high-risk settings such as health care because existing regulations simply cannot keep up with the pace of technological advancements. Work may also need to involve drawing on ongoing efforts in other industries where these challenges have begun to be addressed. Health care robotics is an emerging field that will need inclusive, designated working groups at national and international levels because many functions are patient- and staff-facing and humans and machines need to coexist and collaborate in high-risk environments.

**Table 3:** Sociotechnical challenges identified with suggested strategies.

Identified challenge	Suggested strategy
No clear pull from professionals and patients	Establish an accessible empirical evidence base associated with specific functionalities; communication of benefits and challenges
Appearance of robots	Closer working relationships between developers, psychologists, users, and human-centered design specialists
Changes to the way health care work is organized and distributed	Prospective longitudinal evaluation of the implementation, adoption, and optimization of technologies
New ethical and legal challenges	Development of new ethical and regulatory frameworks that are flexible enough to keep up with changing environments and robotic functionality

Robotics in designated controlled environments (such as service robots) are likely to be less problematic and bring the highest gains in the short term because they present a limited number of sociotechnical challenges compared with applications that blur social and technical dimensions (eg, humanoids).

### Conclusions

Sociotechnical challenges surrounding the implementation of robotics in health care settings are significant, although these are likely to vary with different robotic applications and in different cultural contexts. These challenges need to be anticipated and, if possible, proactively addressed. Health care settings are characterized by their care work; the provocation is to preserve and intensify or augment this within an increasingly automated and technological environment. This can only be done if we anticipate challenges associated with new technologies and systematically address them as we integrate them within existing social orders. Our research should be seen as a stepping stone to stimulate wider discussions surrounding these challenges. It can also help to guide health care organizations and policy makers as they make important strategic decisions associated with purchasing, developing, and deploying robotic applications.

## Abbreviations

HIT: health information technology
EHR: electronic health record
